# Comparison of the Response to an Electronic Versus a Traditional Informed Consent Procedure in Terms of Clinical Patient Characteristics: Observational Study

**DOI:** 10.2196/54867

**Published:** 2024-07-11

**Authors:** Anna G M Zondag, Marieke J Hollestelle, Rieke van der Graaf, Hendrik M Nathoe, Wouter W van Solinge, Michiel L Bots, Robin W M Vernooij, Saskia Haitjema, UCC-CVRM study group

**Affiliations:** 1 Central Diagnostic Laboratory University Medical Center Utrecht Utrecht University Utrecht Netherlands; 2 Julius Center for Health Sciences and Primary Care University Medical Center Utrecht Utrecht University Utrecht Netherlands; 3 Department of Cardiology University Medical Center Utrecht Utrecht University Utrecht Netherlands; 4 Department of Nephrology and Hypertension University Medical Center Utrecht Utrecht Netherlands; 5 See Acknowledgements

**Keywords:** informed consent, learning health care system, e-consent, cardiovascular risk management, digital health, research ethics

## Abstract

**Background:**

Electronic informed consent (eIC) is increasingly used in clinical research due to several benefits including increased enrollment and improved efficiency. Within a learning health care system, a pilot was conducted with an eIC for linking data from electronic health records with national registries, general practitioners, and other hospitals.

**Objective:**

We evaluated the eIC pilot by comparing the response to the eIC with the former traditional paper-based informed consent (IC). We assessed whether the use of eIC resulted in a different study population by comparing the clinical patient characteristics between the response categories of the eIC and former face-to-face IC procedure.

**Methods:**

All patients with increased cardiovascular risk visiting the University Medical Center Utrecht, the Netherlands, were eligible for the learning health care system. From November 2021 to August 2022, an eIC was piloted at the cardiology outpatient clinic. Prior to the pilot, a traditional face-to-face paper-based IC approach was used. Responses (ie, consent, no consent, or nonresponse) were assessed and compared between the eIC and face-to-face IC cohorts. Clinical characteristics of consenting and nonresponding patients were compared between and within the eIC and the face-to-face cohorts using multivariable regression analyses.

**Results:**

A total of 2254 patients were included in the face-to-face IC cohort and 885 patients in the eIC cohort. Full consent was more often obtained in the eIC than in the face-to-face cohort (415/885, 46.9% vs 876/2254, 38.9%, respectively). Apart from lower mean hemoglobin in the full consent group of the eIC cohort (8.5 vs 8.8; *P*=.0021), the characteristics of the full consenting patients did not differ between the eIC and face-to-face IC cohorts. In the eIC cohort, only age differed between the full consent and the nonresponse group (median 60 vs 56; *P*=.0002, respectively), whereas in the face-to-face IC cohort, the full consent group seemed healthier (ie, higher hemoglobin, lower glycated hemoglobin [HbA_1c_], lower C-reactive protein levels) than the nonresponse group.

**Conclusions:**

More patients provided full consent using an eIC. In addition, the study population remained broadly similar. The face-to-face IC approach seemed to result in a healthier study population (ie, full consenting patients) than the patients without IC, while in the eIC cohort, the characteristics between consent groups were comparable. Thus, an eIC may lead to a better representation of the target population, increasing the generalizability of results.

## Introduction

The use of electronic informed consent (eIC) procedures in clinical research is increasing due to several benefits, including increased enrollment and improved efficiency, by reducing the need for on-site research staff and the associated paperwork [1-3]. eICs have the potential to improve the patient experience (eg, patient understanding and confidence) of the informed consent (IC) process, in part because of the ability to include digital multimedia [4,5]. Alternatively, concerns were raised about whether eIC forms are easily accessible to an elderly population and those with limited digital literacy [6], making it more difficult to assess whether the patient has fully understood the IC form, one of the requirements for a valid IC [7]. Standardized best practices for eIC procedures are still lacking [5,8].

In 2014, the University Medical Center (UMC) Utrecht in the Netherlands initiated the Utrecht Cardiovascular Cohort-CardioVascular Risk Management (UCC-CVRM) as a learning health care system (LHS). The UCC-CVRM LHS aims to improve uniform assessment and registration of cardiovascular risk indicators, based on Dutch national guidelines, in electronic health records (EHRs) for all patients referred to the UMC Utrecht for cardiovascular evaluation [9]. In an LHS, care and research are integrated in such a way that health care activities are continuously analyzed and the knowledge gained from these analyses is used to improve care by changing health care practices [10]. In the case of UCC-CVRM LHS, a traditional face-to-face IC procedure was used for blood sample storage in a biobank and the reuse of routine care data for scientific research purposes including linkage of data from EHRs to national registries [9]. In 2020, during the COVID-19 pandemic, the UCC-CVRM steering committee evaluated the study including the IC procedure [11,12]. In short, less than half of the patients who were invited to participate, 41.5% (2378/5730), provided written IC [12]. Next, patients who did consent differed in clinical characteristics from those nonconsenting or nonresponding, clearly leading to a selection of patients not representable for all eligible patients. For example, consenting patients had a lower cardiovascular disease burden than nonconsenting patients [11,12]. In addition, structured registration of cardiovascular risk management (CVRM) indicators in the EHR was worse compared with consenting patients. This selection is detrimental to an LHS, as the population included in the LHS may be less representative of the target population as a whole [11,12]. Finally, eligible patients were not invited, mainly because of the time-consuming and unsustainable IC procedure due to changes in personnel and changes in priorities during peak periods (eg, the COVID-19 pandemic) [13].

Therefore, the UCC-CVRM steering committee decided to alter the approach. Identification of eligible patients for the LHS was to be automated and CVRM data, to be assessed regularly in patients at higher cardiovascular risk, were extracted from structured fields in the EHR. To still enable the linkage of this patient information to data from national registries, general practitioners (GPs), and other hospitals, an eIC procedure was piloted. This study aims to evaluate the eIC procedure by studying the response to the IC form. In addition, we aim to assess whether the change in the IC procedure leads to a different study population by investigating potential differences in clinical characteristics between the response categories of the eIC compared with the former face-to-face IC procedure.

## Methods

We used the STROBE (Strengthening the Reporting of Observational Studies in Epidemiology) statement as a reporting guideline for this study.

### Study Setting

**Figure 1:**
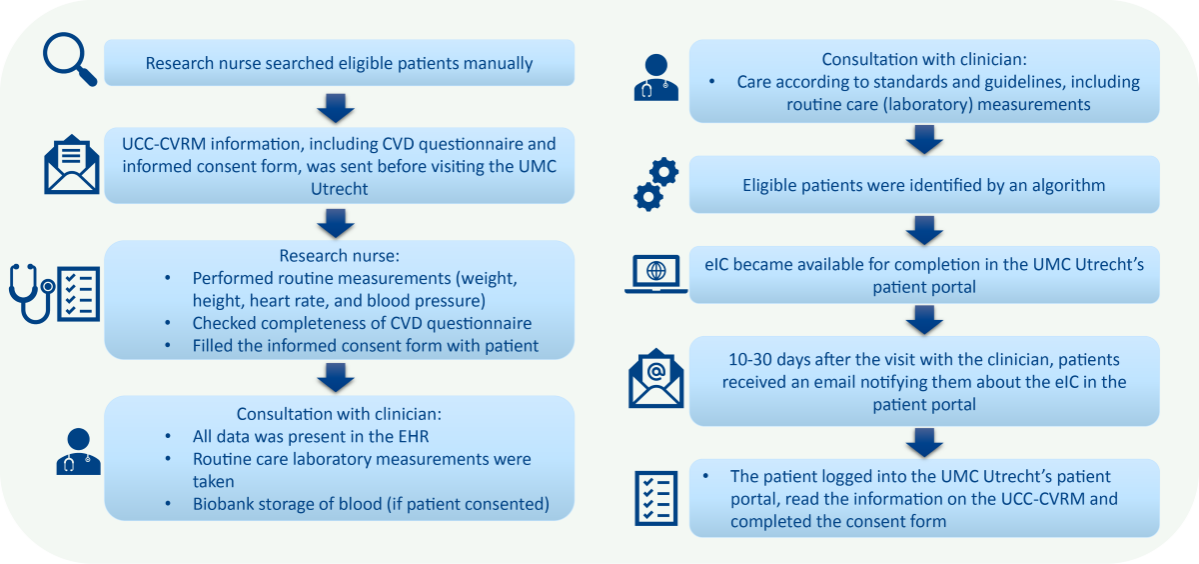
Informed consent procedure of the face-to-face UCC-CVRM (left) and the eIC pilot (right). CVD: cardiovascular disease; EHR: electronic health record; eIC: electronic informed consent; UCC-CVRM: Utrecht Cardiovascular Cohort-CardioVascular Risk Management; UMC: University Medical Center.

Patients visiting the UMC Utrecht for the first time for the evaluation of cardiovascular disease or risk factors were eligible for inclusion in the UCC-CVRM LHS. The full rationale of UCC-CVRM has been described elsewhere [9]. The eIC pilot was conducted between November 2021 and August 2022. During this period, all patients (18 years and older) visiting the cardiology outpatient clinic for first-time evaluation automatically received an email. This email notified these patients about UCC-CVRM and the associated eIC form that was available for completion in the UMC Utrecht patient portal. The IC procedures of the face-to-face IC and eIC are illustrated in Figure 1. The full details eIC form presented to the patients in the patient portal are included in Multimedia Appendix 1.

In both the face-to-face IC and the eIC forms, IC was asked for linkage with national registries, GPs, and other hospitals through the following two statements: (1) I consent to future requests to link with various international or national registries, such as the Central Bureau of Statistics (also known as Statistics Netherlands) where all causes of death are registered, the Dutch Cancer Registration (NKR), where all people with cancer are registered, the National Basic Registration of Hospital Care (LBZ), where all hospital admissions are registered, the Foundation for Pharmaceutical Statistics (SFK), where all people who use medicines are registered, and other regional and national registries. (2) I consent to the retrieval of my medical information from my GP, my pharmacy, and any other hospitals where I have been treated in the past.

### Data Collection

We collected data from all patients aged 18 years or older referred to a cardiology outpatient clinic. We used the Utrecht Patient Oriented Database to collect data from the patients who participated in the eIC pilot, referred to as the “eIC cohort.” The Utrecht Patient Oriented Database comprises data on, among others, patient characteristics and laboratory tests for all patients treated at the UMC Utrecht since 2004 [14]. We collected routine care data related to the patient’s demographics and cardiovascular risk, namely blood pressure, BMI, and laboratory measurements (serum lipids, glycated hemoglobin [HbA_1c_], hemoglobin, and renal function). These data were also collected for the cardiology patients who were invited during the period in which a face-to-face IC procedure was in place, referred to as the “face-to-face IC cohort.” From the face-to-face IC cohort, only patients invited up until December 31, 2019, were included because the COVID-19 pandemic significantly hampered the face-to-face IC procedure.

### Variables

All measurements were extracted from structured fields in the EHR. Blood pressure values were extracted from the EHR ±7 days from the date of the visit at the cardiology outpatient clinic. For other measurements, the closest value, within ±21 days of the visit date, was extracted. If no measurements were found within these cutoffs, the measurement was considered missing. An overview of missingness per variable is added as Multimedia Appendix 2. Age was calculated by subtracting the date of the visit from the patient’s date of birth. The estimated glomerular filtration rate was calculated using the Chronic Kidney Disease Epidemiology Collaboration equation and used as a measure of renal function [15].

### Data Analyses

We presented the yield for both the eIC and face-to-face IC cohort as counts and the percentages of patients who, (1) consented to the linkage of their data with their GP, pharmacy, and other hospitals, and linkage with national registries (ie, “full consent”); (2) did not consent for the linkage of their data with their GP, pharmacy, and other hospitals, nor to the linkage with national registries (ie, “nonconsent”); (3) consented to only 1 of the 2; and (4) the percentage of patients who did not respond at all or provided an answer for only 1 of the 2 statements (ie, “nonresponse”). Due to the limited number of observations in some IC response groups (n<25), especially in the eIC cohort, other than the full consent (n=1291) and nonresponse (n=1477) group, further analyses were restricted to the full consent and nonresponse group only.

To assess differences in patient groups, characteristics of the patients with full consent were stratified by cohort (ie, eIC cohort vs face-to-face IC cohort). As a supplement, we also explored the characteristics of the nonresponders by cohort. Finally, we assessed the differences in patient characteristics between the response categories within each cohort (ie, full consent versus nonresponse).

Clinical characteristics were presented as means with corresponding SDs, medians with corresponding interquartile ranges, or counts and percentages, as appropriate. To quantify differences in characteristics between cohorts, or response categories within cohorts, we performed multivariable linear regression analyses, adjusted for age, categorized into 4 categories with an approximately equal number of observations (18 to 47, 48 to 60, 61 to 70, and 71 to 95 years old), and sex. The assumptions of linear regression (eg, approximate normal distribution of the error terms, homoscedasticity of errors) were assessed. Where needed, we used the Box-Cox method to estimate the most appropriate transformation of the dependent variable to stabilize the variance and improve the accuracy of our estimations [16]. Similarly, multivariable linear regression was used to assess the difference in age (as a continuous variable) between groups, adjusted for sex. Multivariable logistic regression was used to assess the difference in sex between the groups (ie, between the 2 cohorts and between the response categories within each cohort), adjusted for age (categorized into 4 categories with an approximately equal number of observations). As a sensitivity analysis, we repeated the adjusted regression analyses with age as a continuous variable, to assess whether the categorization led to different results.

We used the Bonferroni correction to reduce the risk of a type I error resulting from the multiple tests [17]. Thus, the α that we considered as cutoff, .05, was divided by the number of analyses (N=13) performed per comparison. Therefore, a *P* value ≤.0038 was considered statistically significant.

All statistical analyses were performed using R software (version 4.0.5; The R Foundation) [18].

### Ethical Considerations

We obtained an additional ethical waiver (number 19/641) from the Research Ethics Committee Utrecht to examine the characteristics of patients in all IC response categories. Patients who objected to the use of their clinical data for research purposes via the UMC Utrecht opt-out procedure were excluded from this study. Data were pseudonymized and the patients did not receive any compensation for their participation in this study.

## Results

### Yield of the IC Procedure

In total, 3139 patients participated in this study, of whom 885 (28.2%) participated in the eIC pilot cohort and 2254 (71.8%) in the face-to-face IC cohort (Multimedia Appendix 3). Of all patients from the eIC cohort, 49.9% (442/885) completed the eIC form, 50.1% (443/885) did not respond. Of all patients who completed the eIC form, we obtained full consent for linkage with GPs, hospitals, and national registries from 93.9% (415/442) of the patients. In the face-to-face IC cohort, 54.1% (1220/2254) of all patients completed the IC form, and 45.9% (1034/2254) patients did not respond. The percentage of responding patients with full consent was higher in the eIC cohort as compared with the face-to-face IC cohort (415/442, 93.9% vs 876/1220, 71.8%, respectively).

### Differences in Characteristics Between the eIC and Face-to-Face Cohorts

Overall, fully consenting patients had similar patient characteristics (Table 1). Adjusted for sex and age, the eIC cohort had lower hemoglobin levels and higher HbA_1c_ levels than the face-to-face IC cohort, reaching the multiple testing threshold for statistical significance for hemoglobin (*P*=.0021).

Similarly, we compared the (clinical) characteristics of the nonresponding patients between cohorts, shown in Multimedia Appendix 4. Adjusted for sex, the nonresponders of the eIC cohort were significantly younger and had, adjusted for age and sex, lower c-reactive protein values than the nonresponders of the face-to-face cohort. No other differences were observed.

**Table 1:** Differences between patients with full consent by cohort, adjusted for age and sex.

Variable		Full consent					*P* value
		eIC^a^ (n=415)		F2F IC^b^ (n=876)			
Age (years), median (IQR)		60.0 (48.0-70.0)		61.0 (50.0-69.0)		.2529	
**Sex, n (%)**							
	Male	237 (57.1)	476 (54.3)		—^c^		
	Female	178 (42.9)	400 (45.7)		.3239		
BMI (kg/m^2^), mean (SD)		26.6 (5.2)		26.7 (5.7)		.8981	
SAP^d^ (mm Hg), mean (SD)		132.1 (19.4)		137.6 (19.6)		.0586	
Hemoglobin (mmol/L), mean (SD)		8.5 (1.4)		8.8 (0.9)		.0021	
HbA_1c_^e^ (mmol/mol), median (IQR)		37.5 (34.0-44.0)		37.0 (34.0-40.0)		.0454	
Cholesterol (mmol/L), mean (SD)		4.8 (1.2)		5.1 (1.3)		.1266	
HDL^f^-cholesterol (mmol/L), mean (SD)		1.3 (0.4)		1.4 (0.4)		.0676	
LDL^g^-cholesterol (mmol/L), mean (SD)		2.7 (1.1)		2.9 (1.1)		.1086	
Triglycerides (mmol/L), median (IQR)		1.7 (1.1-2.6)		1.6 (1.0-2.1)		.3023	
CRP^h^ (mg/L), median (IQR)		2.0 (0.5-10.0)		2.6 (1.1-8.5)		.6666	
Creatinine (µmol/L), median (IQR)		76.0 (64.2-94.0)		74.0 (64.0-88.0)		.7760	
eGFR CKD-EPI^i^ (mL/min/1.73 m^2^), mean (SD)		83.3 (23.1)		84.5 (22.3)		.7068	

^a^eIC: electronic informed consent.

^b^F2F IC: face-to-face informed consent.

^c^Reference group.

^d^SAP: systolic arterial blood pressure.

^e^HbA_1c_: glycated hemoglobin.

^f^HDL: high-density lipoprotein.

^g^LDL: low-density lipoprotein.

^h^CRP: c-reactive protein.

^i^eGFR CKD-EPI: estimated glomerular filtration rate calculated using the Chronic Kidney Disease Epidemiology Collaboration equation.

### Differences in Characteristics Between Response Categories

Within each cohort, we assessed whether there were differences in characteristics between the response categories (ie, full consent vs nonresponse). In the eIC cohort, the nonresponse group was significantly younger than the full consent group (Table 2). Other than that, the clinical characteristics of the full consent group were similar to those of the nonresponse group.

More differences were found between the response categories of the face-to-face IC cohort. Adjusted for age and sex, patients in the full consent group had higher hemoglobin, but lower HbA_1c_ and c-reactive protein values than the nonresponse group (Table 3).

**Table 2:** Differences between the response categories in the electronic informed consent cohort, adjusted for age and sex.

Variable		Full consent (n=415)	Nonresponse (n=443)	*P* value
Age (years), median (IQR)		60.0 (48.0-70.0)	56.0 (28.0-72.0)	.0002
**Sex, n (%)**				
	Male	237 (57.1)	222 (50.1)	—^a^
	Female	178 (42.9)	221 (49.9)	.0420
BMI (kg/m^2^), mean (SD)		26.6 (5.2)	26.0 (4.9)	.3673
SAP^b^ (mm Hg), mean (SD)		132.1 (19.4)	130.4 (19.6)	.4168
Hemoglobin (mmol/L), mean (SD)		8.5 (1.4)	8.4 (1.3)	.2397
HbA_1c_^c^ (mmol/mol), median (IQR)		37.5 (34.0-44.0)	37.5 (34.0-40.2)	.1940
Cholesterol (mmol/L), mean (SD)		4.8 (1.2)	4.6 (1.5)	.2852
HDL^d^-cholesterol (mmol/L), mean (SD)		1.3 (0.4)	1.2 (0.5)	.3371
LDL^e^-cholesterol (mmol/L), mean (SD)		2.7 (1.1)	2.6 (0.9)	.9304
Triglycerides (mmol/L), median (IQR)		1.7 (1.1-2.6)	1.4 (1.1-2.0)	.4167
CRP^f^ (mg/L), median (IQR)		2.0 (0.5-10.0)	3.0 (0.5-12.0)	.5922
Creatinine (µmol/L), median (IQR)		76.0 (64.2-94.0)	79.0 (64.0-100.5)	.0897
eGFR CKD-EPI^g^ (mL/min/1.73 m^2^), mean (SD)		83.3 (23.1)	82.0 (30.7)	.1103

^a^Reference group.

^b^SAP: systolic arterial blood pressure.

^c^HbA_1c_: glycated hemoglobin.

^d^HDL: high-density lipoprotein.

^e^LDL: low-density lipoprotein.

^f^CRP: c-reactive protein.

^g^eGFR CKD-EPI: estimated glomerular filtration rate calculated using the Chronic Kidney Disease Epidemiology Collaboration equation.

**Table 3:** Differences between the response categories of the face-to-face informed consent cohort, adjusted for age and sex.

Variable		Full consent (n=876)	Nonresponse (n=1034)	*P* value
Age, median (IQR)		61.0 (50.0-69.0)	61.0 (48.0-71.0)	.9461
**Sex, n (%)**				
	Male	476 (54.3)	552 (53.4)	—^a^
	Female	400 (45.7)	482 (46.6)	.7859
BMI (kg/m^2^), mean (SD)		26.7 (5.7)	26.2 (5.5)	.1063
SAP^b^ (mm Hg), mean (SD)		137.6 (19.6)	136.3 (22.0)	.1093
Hemoglobin (mmol/L), mean (SD)		8.8 (0.9)	8.3 (1.2)	<.0001
HbA_1c_^c^ (mmol/mol), median (IQR)		37.0 (34.0-40.0)	38.0 (34.0-42.0)	.0001
Cholesterol (mmol/L), mean (SD)		5.1 (1.3)	5.0 (1.4)	.4493
HDL^d^-cholesterol (mmol/L), mean (SD)		1.4 (0.4)	1.3 (0.4)	.0898
LDL^e^-cholesterol (mmol/L), mean (SD)		2.9 (1.1)	2.9 (1.1)	.2754
Triglycerides (mmol/L), median (IQR)		1.6 (1.0-2.1)	1.6 (1.0-2.4)	.0435
CRP^f^ (mg/L), median (IQR)		2.6 (1.1-8.5)	8.1 (2.0-38.2)	<.0001
Creatinine (µmol/L), median (IQR)		74.0 (64.0-88.0)	75.0 (63.0-92.0)	.4361
eGFR CKD-EPI^g^ (mL/min/1.73 m^2^), mean (SD)		84.5 (22.3)	81.3 (29.0)	.0946

^a^Reference group.

^b^SAP: systolic arterial blood pressure.

^c^HbA_1c_: glycated hemoglobin.

^d^HDL: high-density lipoprotein.

^e^LDL: low-density lipoprotein.

^f^CRP: c-reactive protein.

^g^eGFR CKD-EPI: estimated glomerular filtration rate calculated using the Chronic Kidney Disease Epidemiology Collaboration equation.

### Sensitivity Analysis

We repeated the regression analyses adjusted for sex and age. In these regression analyses, age was maintained continuous instead of categorized, to assess whether the categorization of age led to different results. The results were similar (Multimedia Appendix 5).

## Discussion

### Principal Results

We showed that by using an eIC in an LHS, patients more often provided full consent to link their data to national registries, GPs, and other hospitals compared with a face-to-face IC procedure. The clinical characteristics of patients with full consent remained largely similar after changing the IC procedure to an eIC. Except for age, we did not find any differences between the response categories of the eIC cohort, whereas in the face-to-face cohort, several differences were found. These differences potentially suggest a higher (cardiovascular) disease burden in the nonresponse group compared with the full consent group, indicative of a potentially more pronounced selection in the face-to-face approach.

A possible explanation for the differences in characteristics between the response categories in the face-to-face cohort is that patients may have been too ill or frail to attend the physical appointment with the research nurse to discuss and sign the IC form, resulting in nonresponse. The inability to attend the appointment was probably less of an issue in the eIC cohort, as patients were able to access the eIC form remotely. The finding suggests that the use of eIC results in a study population (ie, those who give full consent) that is more representative of the full target population. Our findings agree with a previous study showing that providing computer-based clinical study information leads to more willingness to participate [19], as the increased willingness to participate is consistent with the higher full consent rates found in the eIC group compared with the face-to-face IC group in our study.

### Frequently Mentioned Challenges of eICs in the Literature

Concerns have been raised about whether consent given via an eIC is truly an “informed” consent [8]. According to the principles of the Declaration of Helsinki [7], potential participants must be adequately informed about various aspects of the study, such as its purpose, sources of funding, the anticipated benefits and potential risks, and the right to refuse or withdraw consent to participate without giving a reason [7]. According to previous research, comprehension assessment is more challenging when an eIC procedure is used as there is no direct interaction between the potential participant and researcher [6]. As a result, patients might provide consent without fully understanding what they are consenting to, or, conversely, patients may be less likely to consent because of the lack of personal interaction with the researcher or clinician, especially those who were already doubtful about participating in the first place. However, our findings indicate that the latter might not have been the case in our pilot study, as we observed a higher percentage of patients with full consent in the eIC cohort compared with the face-to-face IC cohort.

Another frequently mentioned concern is that studies using an eIC procedure could become inaccessible to patients who lack the digital literacy needed to access and understand the eIC form [6]. In 2021, the Netherlands had the highest percentage (ie, 79%) of 17- to 74-year-olds with at least basic digital skills in Europe [20]. Therefore, incomprehension of the eIC due to limited digital literacy may appear less of an issue in our study. However, the percentage of persons with basic digital skills varied considerably by age, with older people being less literate [20]. A sensitivity analysis showed that the age distribution of responding patients was similar between the eIC and the face-to-face approach (Multimedia Appendix 6), indicating that the eIC was not less accessible than the face-to-face IC for certain age groups. However, accessibility may be an issue for geriatric patients, who are generally older than cardiology patients and often have geriatric syndromes that sometimes affect comprehension and literacy [21]. These syndromes generally make it difficult to obtain IC from the elderly [21]. eIC could, therefore, also be seen as an opportunity. Unlike paper-based ICs, multiple formats can be used to inform the patient about the purpose of the eIC and to provide technical support, for example, by using instructional videos or audio. The use of multiple formats in IC forms for the elderly has been recommended by, among others, Barron et al [22]. Furthermore, UCC-CVRM’s eIC form is available in UMC Utrecht’s long-existing patient portal. In the portal, patients have the opportunity to, among others, ask questions to their clinician via an e-consult, which can be used if parts of the eIC are unclear [23]. Another possibility would be a hybrid format, allowing patients who prefer correspondence by regular mail to respond using a paper-based IC form. However, it is questionable whether this would be helpful and it would negate the positive aspects of the eIC highlighted in this study (eg, less pronounced selection).

### Legislation and Regulation Regarding eIC

Since July 2022, eICs have been permitted in the Netherlands when certain conditions are met [24]. A total of 6 conditions are described in the guideline written by the Central Committee on Research Involving Human Subjects (Centrale Commissie Mensgebonden Onderzoek) and the Dutch Association of Medical Research Ethics Committees (Nederlandse Vereniging voor Medisch-Ethische toetsingscommissies) [25]. The most important conditions are (1) eIC must be appropriate for the study, meaning that the study is associated with low potential risk and burden for the patient, (2) the eIC process must be sufficiently reliable and confidential, guaranteed by an electronic system that is compliant to the Dutch General Data Protection Regulation (UAVG in Dutch) and ensures the validity of the electronic signatures, and (3) the eIC procedure must be described in the study protocol [24,25]. The implementation of an eIC seems appropriate in the case of the UCC-CVRM, as no potential risk or burden for the patient is involved. Furthermore, in the eIC of the UCC-CVRM, data security, identity verification, and the validity of the electronic signature are ensured by the Dutch digital ID, an identification method for accessing web-based services [26]. Regarding the third condition, an amendment to the UCC-CVRM approach, including the eIC, was submitted and approved by the Research Ethics Committee.

### Clinical Implications

Based on the results of our study, the use of eIC to obtain IC might be a sustainable and adequate way to enable researchers to link with national registries, GPs, and other hospitals. The use of the eIC seemed to have resulted in a population with consent that is more similar to the target population compared with the face-to-face IC, which is of great importance in an LHS. Results from the LHS would be more generalizable to the target population, namely to all patients at higher cardiovascular risk. Yet, one may argue whether ≈50% response to both the electronic and face-to-face IC for an LHS approach is sufficient. In addition, it should be noted that the extractability of CVRM indicators from structured fields in the EHR was much lower in the eIC cohort compared with the face-to-face IC cohort. Groenhof et al [13] showed that the former, protocolized, face-to-face UCC-CVRM approach led to more systematic registration of the cardiovascular risk profile in the EHR, which had a positive effect on CVRM guideline adherence in consenting patients, compared with the situation before UCC-CVRM was introduced [13]. The substantial missingness in the eIC cohort of our study may suggest that these improvements are at risk when the approach is automated, as deviations from the initial protocol are made, potentially leading to suboptimal CVRM in clinical care.

Exploring the views and experiences of patients could help to further improve the eIC form. Therefore, we recommend further qualitative research into the accessibility and understandability of eICs used for similar purposes and in similar settings as the UCC-CVRM LHS from a patient’s perspective.

### Strengths and Limitations

To the best of our knowledge, we are among the first to investigate the differences in clinical patient characteristics between response categories of an eIC compared with those of a traditional face-to-face IC, specifically in the context of a cardiovascular LHS in a large sample of patients. Our uniqueness, however, limits the ability to compare our findings to the literature, as most research on eIC has focused on user perspectives, experiences, and the ethical considerations of eICs. For example, Chen et al [5] showed that in most included studies, participants had a better understanding of the information when using an eIC compared with a traditional paper-based face-to-face IC, while others found no difference [5]. Nevertheless, they [5] and others [2,6,27] indicated that face-to-face interaction should remain part of the IC process, especially for more complex and higher-risk studies. However, as the UCC-CVRM LHS is not a complex or high-risk study, the face-to-face interaction may be less necessary. Furthermore, the nonresponders in the eIC cohort may not be fully comparable to the nonresponders in the face-to-face IC cohort because, in the eIC cohort, patients received the eIC after their appointment at the cardiology outpatient clinic, whereas in the face-to-face IC cohort, cardiology patients were identified as eligible and received information about the UCC-CVRM LHS prior to their appointment. This means that patients who, for example, canceled their appointment at the last minute would still be included in the face-to-face cohort as nonresponders. It may be that patients who did not attend their appointment at all had different characteristics to those who attended but did not respond to the eIC, potentially affecting the validity of the comparisons made. Finally, the eIC form was piloted in the patient population of the cardiology outpatient clinic only. Although our results indicated that there were only minor differences (ie, hemoglobin) between patients providing full consent using the eIC compared with the face-to-face IC, it remains to be seen whether this would still be the case after implementation of the eIC in other clinical departments.

### Conclusions

To conclude, our findings suggest that using an eIC may lead to a better representation of the target population by consenting patients. This increases the generalizability of results from studies using the data collected within the LHS from consenting patients.

## Appendix

Multimedia Appendix 1 The electronic informed consent form as presented in the patient portal of the UMC (University Medical Center) Utrecht (translated from Dutch to English).

Multimedia Appendix 2 Missingness per variable in count and percentage, by cohort and informed consent response strata.

Multimedia Appendix 3 Yield (ie, response to the informed consent invitation), by type of informed consent. eIC: electronic informed consent; GP: general practitioner.

Multimedia Appendix 4 Differences between patients who did not respond, by cohort, adjusted for age and sex.

Multimedia Appendix 5 Results of the sensitivity analysis in which age is treated as a continuous variable instead of categorical variable.

Multimedia Appendix 6 Age distribution of patients who completed the informed consent form, stratified by cohort.

## Abbreviations

CVRM: cardiovascular risk management
EHR: electronic health record
eIC: electronic informed consent
GP: general practitioner
HbA1c: glycated hemoglobin
IC: informed consent
LHS: learning health care system
STROBE: Strengthening the Reporting of Observational Studies in Epidemiology
UCC-CVRM: Utrecht Cardiovascular Cohort-Cardiovascular Risk Management
UMC: University Medical Center

## References

[1] Simon CM, Klein DW, Schartz HA (2014). Traditional and electronic informed consent for biobanking: a survey of U.S. biobanks. Biopreserv Biobank.

[2] Skelton E, Drey N, Rutherford M, Ayers S, Malamateniou C (2020). Electronic consenting for conducting research remotely: a review of current practice and key recommendations for using e-consenting. Int J Med Inform.

[3] Phillippi JC, Doersam JK, Neal JL, Roumie CL (2018). Electronic informed consent to facilitate recruitment of pregnant women into research. J Obstet Gynecol Neonatal Nurs.

[4] Boutin NT, Mathieu K, Hoffnagle AG, Allen NL, Castro VM, Morash M, O'Rourke PP, Hohmann EL, Herring N, Bry L, Slaugenhaupt SA, Karlson EW, Weiss ST, Smoller JW (2016). Implementation of electronic consent at a biobank: an opportunity for precision medicine research. J Pers Med.

[5] Chen C, Lee P, Pain KJ, Delgado D, Cole CL, Campion TR (2020). Replacing paper informed consent with electronic informed consent for research in academic medical centers: a scoping review. AMIA Jt Summits Transl Sci Proc.

[6] Yusof MYPM, Teo CH, Ng CJ (2022). Electronic informed consent criteria for research ethics review: a scoping review. BMC Med Ethics.

[7] (2022). Declaration of helsinki - ethical principles for medical research involving human subjects. World Medical Association.

[8] Lunt H, Connor S, Skinner H, Brogden G (2019). Electronic informed consent: the need to redesign the consent process for the digital age. Intern Med J.

[9] Asselbergs FW, Visseren FL, Bots ML, de Borst GJ, Buijsrogge MP, Dieleman JM, van Dinther BG, Doevendans PA, Hoefer IE, Hollander M, de Jong PA, Koenen SV, Pasterkamp G, Ruigrok YM, van der Schouw YT, Verhaar MC, Grobbee DE (2017). Uniform data collection in routine clinical practice in cardiovascular patients for optimal care, quality control and research: the Utrecht cardiovascular cohort. Eur J Prev Cardiol.

[10] Wouters RHP, van der Graaf R, Voest EE, Bredenoord AL (2020). Learning health care systems: highly needed but challenging. Learn Health Syst.

[11] Groenhof TKJ, Mostert M, Lea NC, Haitjema S, de Vries MC, van Dijk WB, Grobbee DE, Asselbergs FW, Bots ML, van der Graaf R (2022). How traditional informed consent impairs inclusivity in a learning healthcare system: lessons learned from the Utrecht cardiovascular cohort. J Clin Epidemiol.

[12] Zondag AGM, Groenhof TKJ, van der Graaf R, van Solinge WW, Bots ML, Haitjema S, UCC-CVRM study group (2023). Asking informed consent may lead to significant participation bias and suboptimal cardiovascular risk management in learning healthcare systems. BMC Med Res Methodol.

[13] Groenhof TKJ, Haitjema S, Lely AT, Grobbee DE, Asselbergs FW, Bots ML, UCC-CVRMUPOD Study groups (2023). Optimizing cardiovascular risk assessment and registration in a developing cardiovascular learning health care system: women benefit most. PLOS Digit Health.

[14] ten Berg MJ, Huisman A, van den Bemt PMLA, Schobben AFAM, Egberts ACG, van Solinge WW (2007). Linking laboratory and medication data: new opportunities for pharmacoepidemiological research. Clin Chem Lab Med.

[15] Levey AS, Stevens LA, Schmid CH, Zhang YL, Castro AF, Feldman HI, Kusek JW, Eggers P, Van Lente F, Greene T, Coresh J, CKD-EPI (Chronic Kidney Disease Epidemiology Collaboration) (2009). A new equation to estimate glomerular filtration rate. Ann Intern Med.

[16] Glen S Box cox transformation: definition, examples. Statistics How To.

[17] VanderWeele TJ, Mathur MB (2019). Some desirable properties of the bonferroni correction: is the bonferroni correction really so bad?. Am J Epidemiol.

[18] (2021). R: a language and environment for statistical computing. R foundation for statistical computing.

[19] Karunaratne AS, Korenman SG, Thomas SL, Myles PS, Komesaroff PA (2010). Improving communication when seeking informed consent: a randomised controlled study of a computer-based method for providing information to prospective clinical trial participants. Med J Aust.

[20] (2022). Dutch digital skills at the top in Europe. CBS.

[21] Cherubini A, Gasperini B (2017). How to increase the participation of older subjects in research: good practices and more evidence are needed!. Age Ageing.

[22] Barron JS, Duffey PL, Byrd LJ, Campbell R, Ferrucci L (2004). Informed consent for research participation in frail older persons. Aging Clin Exp Res.

[23] My UMC Utrecht patient portal. UMC Utrecht.

[24] Consent: on paper or electronic (in Dutch only). CCMO.

[25] (2022). Guide to granting electronic consent for participation in medical-scientific research (in Dutch only). CCMO, NVMETC.

[26] (2024). What is DigiD?. Netherlands Worldwide.

[27] De Sutter E, Zaçe D, Boccia S, Di Pietro ML, Geerts D, Borry P, Huys I (2020). Implementation of electronic informed consent in biomedical research and stakeholders' perspectives: systematic review. J Med Internet Res.

