# Supplementary material for: Comparison of the Response to an Electronic Versus a Traditional Informed Consent Procedure in Terms of Clinical Patient Characteristics: Observational Study
Source: J Med Internet Res. 2024 Jul 11;26:e54867. doi: 10.2196/54867 (PMC11273067; doi:10.2196/54867)
Supplement: Multimedia Appendix 1 [file jmir_v26i1e54867_app1.doc]

**The electronic informed consent form as presented in the patient portal of the UMC Utrecht (translated from Dutch to English)**

Will you help to improve care for future patients?

We use medical data from your medical record to improve care at the UMC Utrecht Cardiovascular Center, and we use residual material, blood or tissue that is preserved after research. We would also like to know how you are doing over time. For example, whether you have been admitted to another hospital and, if so, why. This information is important because we want to learn from every patient. For example, whether a patient has been treated well, whether they were never readmitted elsewhere in the hospital for the same condition, or whether the medicines we prescribed are still being used after a long time. We collect this type of data from various national registries and from your GP, pharmacy or other hospitals. We ask your permission for this.

For more information, we would like to refer you to [www.umcutrecht.nl/ucc](http://www.umcutrecht.nl/ucc)

- request your data from your general practitioner, pharmacy or practitioner elsewhere. This is to know how your health has developed over time.
- request your data from national registries. This is also to see how your health has developed over time. A national registry is, for example, the Dutch Cancer Registry (NKR), where all people who have cancer are registered; or, for example, the Dutch Foundation for Pharmaceutical Statistics (SFK), where all people who use medicines are registered; and/or from, for example, the Central Bureau for Statistics (CBS), where all causes of death are registered; or, for example, the registry where all hospital admissions are recorded, the National Hospital Care Basic Registration (LBZ) etc.

Date informed consent: *dd-mm-yyyy*
