# Supplementary material for: Comparison of the Response to an Electronic Versus a Traditional Informed Consent Procedure in Terms of Clinical Patient Characteristics: Observational Study
Source: J Med Internet Res. 2024 Jul 11;26:e54867. doi: 10.2196/54867 (PMC11273067; doi:10.2196/54867)
Supplement: Multimedia Appendix 2 [file jmir_v26i1e54867_app2.doc]

Table S1. Missingness per variable in count and percentage, by cohort and IC response strata.

|  | eIC | | F2F IC | |
| --- | --- | --- | --- | --- |
| Variable | Full consent (n=415) | Nonresponse (n=443) | Full consent (n=876) | Nonresponse (n=1034) |
| Age, n (%) | 0 (0) | 0 (0) | 0 (0) | 0 (0) |
| Sex, n (%) | 0 (0) | 0 (0) | 0 (0) | 0 (0) |
| BMI (kg/m^2^), n (%) | 267 (64.3) | 329 (74.3) | 71 (8.1) | 341 (33) |
| SAP (mmHg), n (%) | 372 (89.6) | 396 (89.4) | 52 (5.9) | 396 (38.3) |
| Haemoglobin (mmol/L), n (%) | 209 (50.4) | 244 (55.1) | 95 (10.8) | 400 (38.7) |
| HbA1c (mmol/mol), n (%) | 385 (92.8) | 407 (91.9) | 146 (16.7) | 636 (61.5) |
| Cholesterol (mmol/L), n (%) | 336 (81) | 372 (84) | 109 (12.4) | 629 (60.8) |
| HDL-cholesterol (mmol/L), n (%) | 336 (81) | 374 (84.4) | 113 (12.9) | 633 (61.2) |
| LDL-cholesterol (mmol/L), n (%) | 337 (81) | 376 (84.9) | 115 (13.1) | 634 (61.3) |
| Triglycerides (mmol/L), n (%) | 335 (80.7) | 374 (84.4) | 112 (12.8) | 626 (60.5) |
| CRP (mg/L), n (%) | 348 (83.9) | 374 (84.4) | 749 (85.5) | 766 (74.1) |
| Creatinine (µmol/L), n (%) | 205 (49.4) | 248 (56) | 90 (10.3) | 401 (38.8) |
| eGFR CKD-EPI (ml/min/1.73m2), n (%) | 205 (49.4) | 248 (56) | 90 (10.3) | 401 (38.8) |

*Notes:* eIC = electronic informed consent, F2f IC = face-to-face informed consent; n = number; % = percentage BMI = body mass index; SAP = systolic arterial blood pressure; HbA1c = glycated haemoglobin; HDL = high-density lipoprotein; LDL = low-density lipoprotein; CRP = c-reactive protein; eGFR CKD-EPI = estimated glomerular filtration rate calculated using the Chronic Kidney Disease Epidemiology Collaboration equation.
