# Supplementary material for: Comparison of the Response to an Electronic Versus a Traditional Informed Consent Procedure in Terms of Clinical Patient Characteristics: Observational Study
Source: J Med Internet Res. 2024 Jul 11;26:e54867. doi: 10.2196/54867 (PMC11273067; doi:10.2196/54867)
Supplement: Multimedia Appendix 3 [file jmir_v26i1e54867_app3.doc]

Figure S1. Yield (ie, response to the IC invitation), by type of IC. eIC: electronic informed consent; GP: general practitioner.
