# Supplementary material for: Comparison of the Response to an Electronic Versus a Traditional Informed Consent Procedure in Terms of Clinical Patient Characteristics: Observational Study
Source: J Med Internet Res. 2024 Jul 11;26:e54867. doi: 10.2196/54867 (PMC11273067; doi:10.2196/54867)
Supplement: Multimedia Appendix 4 [file jmir_v26i1e54867_app4.doc]

Table S2. Differences between patients that did not respond by cohort, adjusted for age and sex.

| Variable |  | Nonresponse – eIC (n=443) | Nonresponse – F2F IC (n=1034) | *P* value |
| --- | --- | --- | --- | --- |
| Age | median (IQR) | 56.0 (28.0 - 72.0) | 61.0 (48.0 - 71.0) | <.0001 |
| Sex |  |  |  |  |
| - Male | n (%) | 222 (50.1) | 552 (53.4) | ref |
| - Female | n (%) | 221 (49.9) | 482 (46.6) | .4719 |
| BMI (kg/m^2^) | mean (SD) | 26.0 (4.9) | 26.2 (5.5) | .8911 |
| SAP (mmHg) | mean (SD) | 130.4 (19.6) | 136.3 (22.0) | .0601 |
| Haemoglobin (mmol/L) | mean (SD) | 8.4 (1.3) | 8.3 (1.2) | .4333 |
| HbA1c (mmol/mol) | median (IQR) | 37.5 (34.0 - 40.2) | 38.0 (34.0 - 42.0) | .3507 |
| Cholesterol (mmol/L) | mean (SD) | 4.6 (1.5) | 5.0 (1.4) | .0165 |
| HDL-cholesterol (mmol/L) | mean (SD) | 1.2 (0.5) | 1.3 (0.4) | .0221 |
| LDL-cholesterol (mmol/L) | mean (SD) | 2.6 (0.9) | 2.9 (1.1) | .1352 |
| Triglycerides (mmol/L) | median (IQR) | 1.4 (1.1 - 2.0) | 1.6 (1.0 - 2.4) | .2101 |
| CRP (mg/L) | median (IQR) | 3.0 (0.5 - 12.0) | 8.1 (2.0 - 38.2) | .0014 |
| Creatinine (µmol/L) | median (IQR) | 79.0 (64.0 - 100.5) | 75.0 (63.0 - 92.0) | .5845 |
| eGFR CKD-EPI (ml/min/1.73m2) | mean (SD) | 82.0 (30.7) | 81.3 (29.0) | .5142 |

*Notes:* eIC = electronic informed consent; F2F IC = face-to-face informed consent; n = number*; P* = probability value adjusted for age and sex; IQR = interquartile range; SD = standard deviation; BMI = body mass index; SAP = systolic arterial blood pressure; HbA1C = glycated haemoglobin; HDL = high-density lipoprotein; LDL = low-density lipoprotein; CRP = c-reactive protein; eGFR CKD-EPI = estimated glomerular filtration rate calculated using the Chronic Kidney Disease Epidemiology Collaboration equation.
