# Supplementary material for: Comparison of the Response to an Electronic Versus a Traditional Informed Consent Procedure in Terms of Clinical Patient Characteristics: Observational Study
Source: J Med Internet Res. 2024 Jul 11;26:e54867. doi: 10.2196/54867 (PMC11273067; doi:10.2196/54867)
Supplement: Multimedia Appendix 5 [file jmir_v26i1e54867_app5.doc]

Table S3. Differences between patients with full consent by cohort, adjusted for age (continuous) and sex.

| Variable |  | Full consent – eIC  (n=415) | Full consent – F2F IC (n=876) | P value |
| --- | --- | --- | --- | --- |
| Age | median (IQR) | 60.0 (48.0 - 70.0) | 61.0 (50.0 - 69.0) | .2529 |
| Sex |  |  |  |  |
| - Male | n (%) | 237 (57.1) | 476 (54.3) | Ref |
| - Female | n (%) | 178 (42.9) | 400 (45.7) | .2907 |
| BMI (kg/m2) | mean (SD) | 26.6 (5.2) | 26.7 (5.7) | .8911 |
| SAP (mmHg) | mean (SD) | 132.1 (19.4) | 137.6 (19.6) | .0619 |
| Haemoglobin (mmol/L) | mean (SD) | 8.5 (1.4) | 8.8 (0.9) | .0016 |
| HbA1c (mmol/mol) | median (IQR) | 37.5 (34.0 - 44.0) | 37.0 (34.0 - 40.0) | .0503 |
| Cholesterol (mmol/L) | mean (SD) | 4.8 (1.2) | 5.1 (1.3) | .1459 |
| HDL-cholesterol (mmol/L) | mean (SD) | 1.3 (0.4) | 1.4 (0.4) | .0753 |
| LDL-cholesterol (mmol/L) | mean (SD) | 2.7 (1.1) | 2.9 (1.1) | .1203 |
| Triglycerides (mmol/L) | median (IQR) | 1.7 (1.1 - 2.6) | 1.6 (1.0 - 2.1) | .2698 |
| CRP (mg/L) | median (IQR) | 2.0 (0.5 - 10.0) | 2.6 (1.1 - 8.5) | .5652 |
| Creatinine (µmol/L) | median (IQR) | 76.0 (64.2 - 94.0) | 74.0 (64.0 - 88.0) | .8322 |
| eGFR CKD-EPI (ml/min/1.73m2) | mean (SD) | 83.3 (23.1) | 84.5 (22.3) | .4781 |

*Notes:* eIC = electronic informed consent; F2F IC = face-to-face informed consent; n = number*; P* = probability value adjusted for age and sex; % = percentage; IQR = interquartile range; SD = standard deviation; BMI = body mass index; SAP = systolic arterial blood pressure; HbA1C = glycated haemoglobin; HDL = high-density lipoprotein; LDL = low-density lipoprotein; CRP = c-reactive protein; eGFR CKD-EPI = estimated glomerular filtration rate calculated using the Chronic Kidney Disease Epidemiology Collaboration equation.

Table 2. Differences between patients that did not respond by cohort, adjusted for age (continuous) and sex.

| Variable |  | Nonresponse – eIC (n=443) | Nonresponse – F2F IC (n=1034) | *P* value |
| --- | --- | --- | --- | --- |
| Age | median (IQR) | 56.0 (28.0 - 72.0) | 61.0 (48.0 - 71.0) | <.0001 |
| Sex |  |  |  |  |
| - Male | n (%) | 222 (50.1) | 552 (53.4) | Ref |
| - Female | n (%) | 221 (49.9) | 482 (46.6) | .3368 |
| BMI (kg/m^2^) | mean (SD) | 26.0 (4.9) | 26.2 (5.5) | .7706 |
| SAP (mmHg) | mean (SD) | 130.4 (19.6) | 136.3 (22.0) | .0681 |
| Haemoglobin (mmol/L) | mean (SD) | 8.4 (1.3) | 8.3 (1.2) | .5636 |
| HbA1c (mmol/mol) | median (IQR) | 37.5 (34.0 - 40.2) | 38.0 (34.0 - 42.0) | .3812 |
| Cholesterol (mmol/L) | mean (SD) | 4.6 (1.5) | 5.0 (1.4) | .0159 |
| HDL-cholesterol (mmol/L) | mean (SD) | 1.2 (0.5) | 1.3 (0.4) | .0150 |
| LDL-cholesterol (mmol/L) | mean (SD) | 2.6 (0.9) | 2.9 (1.1) | .1160 |
| Triglycerides (mmol/L) | median (IQR) | 1.4 (1.1 - 2.0) | 1.6 (1.0 - 2.4) | .2937 |
| CRP (mg/L) | median (IQR) | 3.0 (0.5 - 12.0) | 8.1 (2.0 - 38.2) | .0023 |
| Creatinine (µmol/L) | median (IQR) | 79.0 (64.0 - 100.5) | 75.0 (63.0 - 92.0) | .4328 |
| eGFR CKD-EPI (ml/min/1.73m2) | mean (SD) | 82.0 (30.7) | 81.3 (29.0) | .1825 |

*Notes:* eIC = electronic informed consent; F2F IC = face-to-face informed consent; n = number*; P* = probability value adjusted for age and sex; % = percentage; IQR = interquartile range; SD = standard deviation; BMI = body mass index; SAP = systolic arterial blood pressure; HbA1C = glycated haemoglobin; HDL = high-density lipoprotein; LDL = low-density lipoprotein; CRP = c-reactive protein; eGFR CKD-EPI = estimated glomerular filtration rate calculated using the Chronic Kidney Disease Epidemiology Collaboration equation.

Table 3. Differences between the response categories in the eIC cohort, adjusted for age (continuous) and sex.

| Variable |  | Full consent (n=415) | Nonresponse (n=443) | *P* value |
| --- | --- | --- | --- | --- |
| Age | median (IQR) | 60.0 (48.0 - 70.0) | 56.0 (28.0 - 72.0) | .0002 |
| Sex |  |  |  |  |
| - Male | n (%) | 237 (57.1) | 222 (50.1) | Ref |
| - Female | n (%) | 178 (42.9) | 221 (49.9) | .0672 |
| BMI (kg/m^2^) | mean (SD) | 26.6 (5.2) | 26.0 (4.9) | .3121 |
| SAP (mmHg) | mean (SD) | 132.1 (19.4) | 130.4 (19.6) | .5304 |
| Haemoglobin (mmol/L) | mean (SD) | 8.5 (1.4) | 8.4 (1.3) | .2941 |
| HbA1c (mmol/mol) | median (IQR) | 37.5 (34.0 - 44.0) | 37.5 (34.0 - 40.2) | .2159 |
| Cholesterol (mmol/L) | mean (SD) | 4.8 (1.2) | 4.6 (1.5) | .2574 |
| HDL-cholesterol (mmol/L) | mean (SD) | 1.3 (0.4) | 1.2 (0.5) | .3018 |
| LDL-cholesterol (mmol/L) | mean (SD) | 2.7 (1.1) | 2.6 (0.9) | .8308 |
| Triglycerides (mmol/L) | median (IQR) | 1.7 (1.1 - 2.6) | 1.4 (1.1 - 2.0) | .3916 |
| CRP (mg/L) | median (IQR) | 2.0 (0.5 - 10.0) | 3.0 (0.5 - 12.0) | .4886 |
| Creatinine (µmol/L) | median (IQR) | 76.0 (64.2 - 94.0) | 79.0 (64.0 - 100.5) | .0909 |
| eGFR CKD-EPI (ml/min/1.73m2) | mean (SD) | 83.3 (23.1) | 82.0 (30.7) | .1009 |

*Notes:* n = number; *P* = unadjusted probability value, corrected for age and sex from a regression model; % = percentage; IQR = interquartile range quartile; SD = standard deviation; BMI = body mass index; SAP = systolic arterial blood pressure; HbA1c = glycated haemoglobin; HDL = high-density lipoprotein; LDL = low-density lipoprotein; CRP = c-reactive protein; eGFR CKD-EPI = estimated glomerular filtration rate calculated using the Chronic Kidney Disease Epidemiology Collaboration equation.

Table 4. Differences between the response categories of the face-to-face IC cohort, adjusted for age (continuous) and sex.

| Variable |  | Full consent (n=876) | Nonresponse (n=1034) | *P* value |
| --- | --- | --- | --- | --- |
| Age | median (IQR) | 61.0 (50.0 - 69.0) | 61.0 (48.0 - 71.0) | .9461 |
| Sex |  |  |  |  |
| - Male | n (%) | 476 (54.3) | 552 (53.4) | Ref |
| - Female | n (%) | 400 (45.7) | 482 (46.6) | .6798 |
| BMI (kg/m^2^) | mean (SD) | 26.7 (5.7) | 26.2 (5.5) | .0627 |
| SAP (mmHg) | mean (SD) | 137.6 (19.6) | 136.3 (22.0) | .0880 |
| Haemoglobin (mmol/L) | mean (SD) | 8.8 (0.9) | 8.3 (1.2) | <.0001 |
| HbA1c (mmol/mol) | median (IQR) | 37.0 (34.0 - 40.0) | 38.0 (34.0 - 42.0) | .0001 |
| Cholesterol (mmol/L) | mean (SD) | 5.1 (1.3) | 5.0 (1.4) | .3553 |
| HDL-cholesterol (mmol/L) | mean (SD) | 1.4 (0.4) | 1.3 (0.4) | .0885 |
| LDL-cholesterol (mmol/L) | mean (SD) | 2.9 (1.1) | 2.9 (1.1) | .2263 |
| Triglycerides (mmol/L) | median (IQR) | 1.6 (1.0 - 2.1) | 1.6 (1.0 - 2.4) | .0680 |
| CRP (mg/L) | median (IQR) | 2.6 (1.1 - 8.5) | 8.1 (2.0 - 38.2) | <.0001 |
| Creatinine (µmol/L) | median (IQR) | 74.0 (64.0 - 88.0) | 75.0 (63.0 - 92.0) | .4707 |
| eGFR CKD-EPI (ml/min/1.73m2) | mean (SD) | 84.5 (22.3) | 81.3 (29.0) | .1491 |

*Notes:* n = number; *P* = unadjusted probability value, corrected for age and sex from a regression model; % = percentage; IQR = interquartile range; SD = standard deviation; BMI = body mass index; SAP = systolic arterial blood pressure; HbA1c = glycated haemoglobin; HDL = high-density lipoprotein; LDL = low-density lipoprotein; CRP = c-reactive protein; eGFR CKD-EPI = estimated glomerular filtration rate calculated using the Chronic Kidney Disease Epidemiology Collaboration equation.
