# Supplementary figures and images for: Comparison of the Response to an Electronic Versus a Traditional Informed Consent Procedure in Terms of Clinical Patient Characteristics: Observational Study
Source: J Med Internet Res. 2024 Jul 11;26:e54867. doi: 10.2196/54867 (PMC11273067; doi:10.2196/54867)

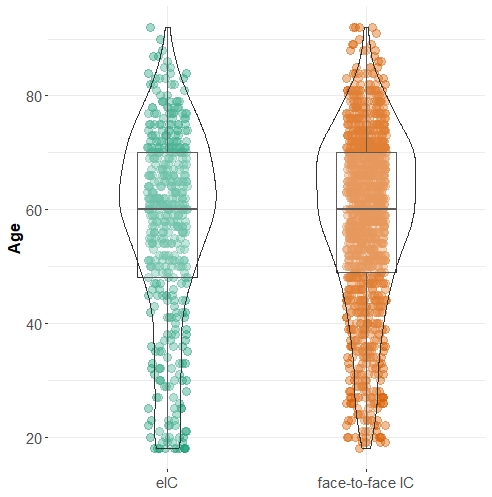


Figure S2. Age distribution of patients that completed the IC form, stratified by cohort.

Supplement: Multimedia Appendix 6 [file jmir_v26i1e54867_app6.doc]
